# Proteomic Changes of Osteoclast Differentiation in Rheumatoid and Psoriatic Arthritis Reveal Functional Differences

**DOI:** 10.3389/fimmu.2022.892970

**Published:** 2022-07-04

**Authors:** Orsolya Tünde Kovács, Eszter Tóth, Olivér Ozohanics, Eszter Soltész-Katona, Nikolett Marton, Edit Irén Buzás, László Hunyady, László Drahos, Gábor Turu, György Nagy

**Affiliations:** ^1^Department of Genetics, Cell- and Immunobiology, Semmelweis University, Budapest, Hungary; ^2^Department of Physiology, Semmelweis University, Budapest, Hungary; ^3^Institute of Organic Chemistry, Eötvös Loránd Research Network, Research Centre for Natural Sciences, Budapest, Hungary; ^4^Department of Biochemistry, Semmelweis University, Budapest, Hungary; ^5^Eötvös Loránd Research Network and Semmelweis University (ELKH-SE) Laboratory of Molecular Physiology, Eötvös Loránd Research Network, Budapest, Hungary; ^6^Department of Radiology, Medical Imaging Centre, Semmelweis University, Budapest, Hungary; ^7^Eötvös Loránd Research Network and Semmelweis University (ELKH-SE) Immune-Proteogenomics Research Group, Budapest, Hungary; ^8^Hungarian Centre of Excellence for Molecular Medicine - Semmelweis University (HCEMM-SU) Extracellular Vesicles Research Group, Budapest, Hungary; ^9^Institute of Enzymology, Eötvös Loránd Research Network, Research Centre for Natural Sciences, Budapest, Hungary; ^10^Department of Rheumatology and Clinical Immunology, Department of Internal Medicine and Oncology, Semmelweis University, Budapest, Hungary; ^11^Heart and Vascular Centre, Semmelweis University, Budapest, Hungary

**Keywords:** mass spectrometry, osteoclast, rheumatoid arthritis, psoriatic arthritis, proteomics

## Abstract

**Background:**

Osteoclasts play a crucial role in the maintenance, repair, and remodeling of bones of the adult vertebral skeleton due to their bone resorption capability. Rheumatoid arthritis (RA) and psoriatic arthritis (PsA) are associated with increased activity of osteoclasts.

**Objectives:**

Our study aimed to investigate the dynamic proteomic changes during osteoclast differentiation in healthy donors, in RA, and PsA.

**Methods:**

Blood samples of healthy donors, RA, and PsA patients were collected, and monocytes were isolated and differentiated into osteoclasts *in vitro* using macrophage colony-stimulating factor (M-CSF) and receptor activator of nuclear factor κB ligand (RANK-L). Mass spectrometry-based proteomics was used to analyze proteins from cell lysates. The expression changes were analyzed with Gene Set Enrichment Analysis (GSEA).

**Results:**

The analysis of the proteomic changes revealed that during the differentiation of the human osteoclasts, expression of the proteins involved in metabolic activity, secretory function, and cell polarity is increased; by contrast, signaling pathways involved in the immune functions are downregulated. Interestingly, the differences between cells of healthy donors and RA/PsA patients are most pronounced after the final steps of differentiation to osteoclasts. In addition, both in RA and PsA the differentiation is characterized by decreased metabolic activity, associated with various immune pathway activities; furthermore by accelerated cytokine production in RA.

**Conclusions:**

Our results shed light on the characteristic proteomic changes during human osteoclast differentiation and expression differences in RA and PsA, which reveal important pathophysiological insights in both diseases.

## 1 Introduction

Bone remodeling is an essential process maintained by osteoclasts, which can uniquely break down bone and calcified cartilage and osteoblasts, which form the new bone (1). Osteoclast precursors are formed from bone marrow-derived monocyte/macrophage progenitor cells when stimulated with M-CSF and in the presence of M-CSF and RANK-L, they differentiate further to mononuclear prefusion osteoclasts, which then fuse into polarized multinuclear osteoclasts. Both RANK-L and M-CSF are essential for the differentiation, activation, and survival of osteoclasts (2–5). Isolating monocytes from blood samples and differentiating them into osteoclasts *in vitro* is a widely used model in human osteoclast studies since they are morphologically and functionally similar to physiological osteoclasts (6, 7). In these studies, adherent conditions are also required besides the application of M-CSF and RANK-L (8). Other methods for obtaining osteoclasts for *in vitro* studies include the differentiation of the murine RAW264.7 cell line or murine bone marrow macrophage (BMM) cultures (9, 10). Lately, human-derived pluripotent stem cells are also being used (11).

Imbalance in the finely regulated osteoblast-osteoclast system plays a central role in the pathogenesis of several metabolic bone and systemic inflammatory diseases. Due to the complex interactions of the bone and the immune system, inflammation may accelerate/increase bone resorption *via* osteoclast activation. Local osteoclast activation is promoted by proinflammatory cytokines released during inflammation (TNF-α, IL-1, IL-6, IL-17, IL-23), by RANK-L produced by lymphocytes and fibroblasts, and by direct cell-cell interactions (12, 13). The production of IL-17 by Th17 lymphocytes is elevated in certain inflammatory conditions, which has a synergic effect with IL-1 and TNF-α (14). Consequently, osteoclasts and synovial fibroblasts in the joint can be activated. Therefore, inflammation is associated with local and systemic bone loss in inflammatory arthropathies e.g. rheumatoid arthritis (RA) or psoriatic arthritis (PsA).

Mass spectrometry (MS)-based proteomics is widely used for the identification and quantification of proteins expressed in diverse biological samples, and to analyze the expression of the cell signaling pathway components. There are a few published articles regarding the proteomics of osteoclasts differentiated from murine precursor cell lines (15–21) and BMM cultures (22). Although the proteomics of isolated monocytes from human peripheral blood was also examined (23–25), as far as we know, there is no published data regarding the proteome of human blood-derived preosteoclasts and osteoclasts and there is little information about the molecular changes during human blood-derived osteoclast differentiation. Also, there is no available information about the differences in protein expression in osteoclasts derived from RA or PsA patients.

In this study, we aimed to analyze proteomic changes during human osteoclast differentiation in healthy donors, RA, and PsA patients. We identify pathways that characterize the protein expression changes during the process and describe key pathways which differ in patients with inflammatory arthropathies. Understanding the characteristics of the proteome changes may help to expand our knowledge about the pathomechanisms involved in these diseases.

## 2 Materials and Methods

### 2.1 Patients

RA and PsA patients’ and healthy donors’ blood was collected in the rheumatology outpatient department of the Semmelweis University in the Hospital of Hospitaller Brothers of St. John of God (Budapest, Hungary). Patients were diagnosed with RA according to the 2010 ACR (American College of Rheumatology)/EULAR (European League Against Rheumatism) classification criteria (26), or with polyarthritic PsA according to the CASPAR (Classification Criteria for Psoriatic Arthritis) classification criteria (27). Based on their DAS-28 (disease activity score calculator for RA) or DAPSA (disease activity in PsA score) disease activity scores, patients had low, moderate, or high disease activity, or were in remission. The national ethics committee approved the study and informed consent was obtained from each individual (approval number: 8490-2/2018/EÜIG). This work was accomplished following the Helsinki Declaration (28).

### 2.2 Chemicals and Reagents

Ficoll, MEM α, L-glutamine, and Penicillin–Streptomycin were obtained from Sigma (St. Louis, MO, USA). FBS, PBS, and trypsin-EDTA solutions were purchased from Gibco Laboratories (Gaithersburg, MD, USA). EasySep was obtained from StemCell Technologies (Vancouver, Canada), rh M-CSF, and rh RANKL were purchased from PeproTech (London, UK).

Acetonitrile, water, formic acid, ammonium-bicarbonate, and sodium deoxycholate were obtained from Merck (Darmstadt, Germany). Methanol was purchased from VWR International (Debrecen, Hungary). Dithiothreitol, iodoacetamide, and TFA were purchased from Thermo Scientific (Waltham, MA, USA), and RapiGest SF surfactant was obtained from Waters (Milford, MA, USA). Trypsin/Lys-C Mix and Trypsin Gold were purchased from Promega Corporation (Madison​, WI, USA). All chemicals, reagents, and solvents were HPLC-MS grade.

### 2.3 *In Vitro* Osteoclast Culture

Human blood samples were collected into vacutainer EDTA tubes (Greiner Bio-One, Mosonmagyarovar, Hungary). PBMCs (peripheral blood mononuclear cells) were isolated *via* a Ficoll density gradient (Sigma, St. Louis, MO, USA). CD14+ cells were isolated from PBMCs using a positive magnetic selection method (EasySep, StemCell Technologies, Vancouver, Canada) (29). After isolation, a third of the monocytes were immediately frozen in liquid nitrogen. Two-thirds of monocytes were cultured (1x10^5^ monocytes per well) in MEM α (Minimum Essential Medium α) (Sigma, St. Louis, MO, USA) with 10% FBS (Gibco Laboratories, Gaithersburg, MD, USA), 1% L-glutamine (Sigma, St. Louis, MO, USA), and 1% Penicillin–Streptomycin (Sigma, St. Louis, MO, USA) at 37°C and 5% CO_2_ in a humidified atmosphere. Monocytes were incubated with 50 ng/mL rh M-CSF (macrophage colony-stimulating factor) for 24 h (PeproTech, London, UK). Thereafter the samples were stimulated with 50 ng/mL of both rh M-CSF and rh RANKL (receptor activator of nuclear factor κB ligand) (PeproTech, London, UK). From then the media was replaced every 3-4 days.

### 2.4 Protein Isolation

On day 1, we froze the monocytes in liquid nitrogen. On days 5 and 9 of culture, differentiating pre-osteoclasts and osteoclast cells were washed in phosphate-buffered saline (PBS, Gibco Laboratories, Gaithersburg, MD, USA) and lysed directly in the culture plate by adding trypsin-EDTA solution (Gibco Laboratories, Gaithersburg, MD, USA), then frozen in liquid nitrogen. Samples were stored at - 80°C for further investigation.

### 2.5 Cell Lysis

Cells stored in the medium were centrifuged, and pellets were washed using an ammonium-bicarbonate buffer. Cells were lysed and proteins were reduced in 50 μL lysis buffer containing 2% sodium deoxycholate, 0.5% RapiGest SF surfactant, and 100 mM dithiothreitol, heated 60°C for 30 min and sonicated as well as vortexed for 10 cycles (10 x 1 min). Protein concentration of the supernatant was measured using NanoDrop™ 1000 (Thermo Scientific, Waltham, MA, USA) as 5-15 mg/mL for most of the samples. If the concentration was >15 mg/mL, an additional 50 μL of lysis buffer was added to the samples.

### 2.6 Proteomic Analysis

Samples containing 2.5 nmol protein were first alkylated and digested with LysC and trypsin for 4 hours, then they were analyzed using a Dionex UltiMate 3000 RSLCnano system (Sunnyvale, CA, USA) coupled with a Maxis II QTOF mass spectrometer (Bruker Daltonik GmbH, Bremen, Germany). Protein identification, quantitation was performed using the Byonic v3.8.18 (https://proteinmetrics.org) and MaxQuant v1.6.17 (https://maxquant.org) software respectively. Detailed methodology – including missing data imputation and differential expression statistics – is described in the methodological supplementary.

### 2.7 Data Analysis

#### 2.7.1 Gene Set Enrichment Analysis (GSEA)

For the analysis, the protein names were first translated to gene names using the BioMart package in R, and with the help of the msigdbr and piano packages, the gene set enrichment was calculated using the https://www.gsea-msigdb.org/gsea/msigdb database. Specifically, we used GO Biological Process and GO Cellular Component ontology gene sets (C5) with ‘fsgea’ geneSetStat parameter.

#### 2.7.2 Plots and Statistics

The plots were created in python using matplotlib (D. Hunter, “Matplotlib: A 2D Graphics Environment”, Computing in Science & Engineering, vol. 9, no. 3, pp. 90-95, 2007.) and seaborn (seaborn: statistical data visualization Journal of Open Source Software) libraries. For the evaluation of the clustering performance to separate the samples based on the selected feature, we calculated the Hubert-Arabie adjusted Rand index (30) using the adjusted_rand_score function from the sklearn library.

## 3 Results

### 3.1 Proteome Changes During the Development of the Osteoclasts

**Figure 1:**
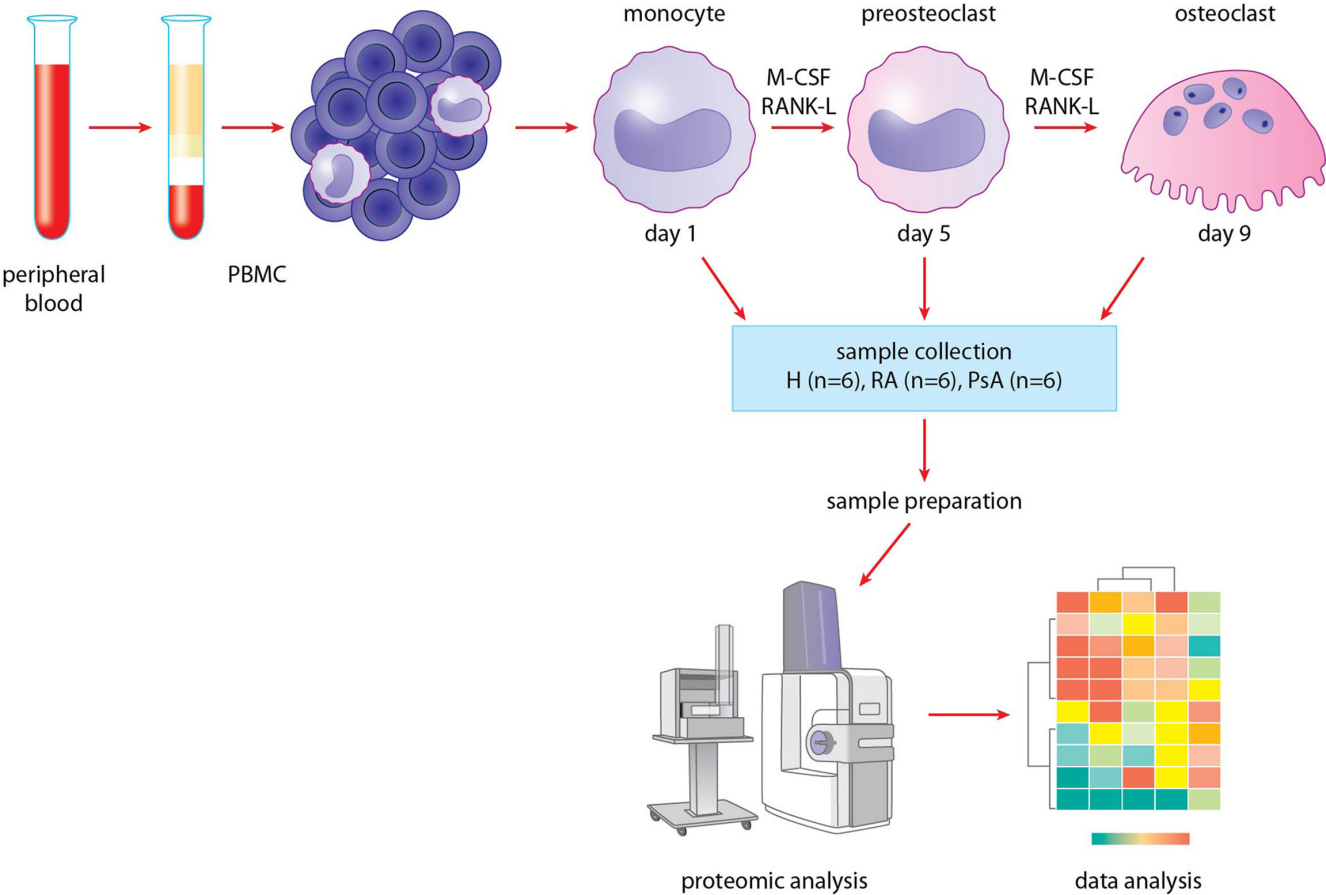
Methodological process. Blood was collected from healthy donors and RA and PsA patients. Monocytes were isolated from PBMCs, then differentiated to preosteoclasts and osteoclasts. Samples were collected, then prepared for proteomic analysis. HPLC-MS/MS analysis was carried out, finally data were analyzed.

To investigate the dynamic proteomic changes during osteoclast differentiation, we used the methodological process shown in **Figure 1**. First, blood samples of six healthy donors and six RA and six PsA patients were collected (**Table 1**). Monocytes were isolated from PBMCs by using a positive magnetic separation, then were cultured as described (31). Cells were differentiated into pre-osteoclasts and osteoclasts, which were collected on days 5 and 9. A total of 18 individual proteomic analyses were performed corresponding to the day 1, 5, and 9 of differentiation using 6-6-6 biological replicates of RA, PsA, and control samples. We identified a total of 1435 proteins (**Supplementary Table 1**) across 18 samples. For further analysis, we used the log2 transformed imputed LFQ values (**Supplementary Table 2**).

**Table 1:** Basic patient characteristics.

Donor	Diagnosis	Sex	Age (year)	DAS-28/DAPSA	Duration of disease (months)	CRP	ESR	RF	anti-CCP	Therapy
1	RA	male	87	2.4	43	2.15	7	–	–	methotrexate
2	RA	female	52	4.6	37	0.3	2	–	–	leflunomide, chloroquine
3	RA	female	67	2.2	75	0.5	18	97	404	methotrexate, chloroquine
4	RA	female	56	2.0	29	1.2	5	41	–	methylprednisolone, methotrexate
5	RA	female	66	0.6	255	0.7	2	128	287	methotrexate
6	RA	female	80	3.9	267	15	38	200	436	methylprednisolone, methotrexate
1	PsA	female	61	3.5	0	11		–	–	–
2	PsA	female	73	3.1	65	4.9	12	–	–	methotrexate
3	PsA	female	54	3.2	141	1.5	11	–	–	methotrexate
4	PsA	male	74	4.0	129	4	2	–	–	methotrexate, chloroquine
5	PsA	female	45	5.0	153	2.99	2	–	–	methotrexate
6	PsA	male	41	5.3	46	2.3	5.3	–	–	methotrexate

Blood samples from 6 patients with RA and 6 with PsA were included in the experiments. Basic characteristics are listed. DAS-28, disease activity score calculator for rheumatoid arthritis; DAPSA, disease activity in psoriatic arthritis score; CRP, C-reactive protein; ESR, erythrocyte sedimentation rate; RF, rheumatoid factor; anti-CCP, anti-cyclic citrullinated peptide autoantibody.

**Figure 2:**
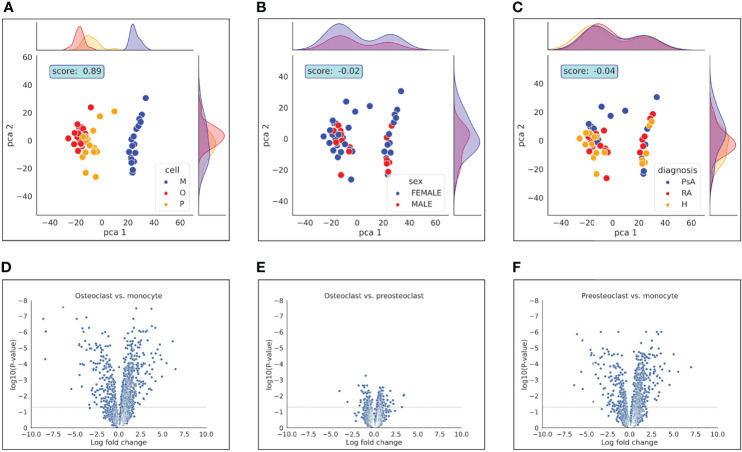
PCA analysis and Volcano plot represent differences between samples. PCA analysis represents the differences between **(A)**: cell types, **(B)**: gender and **(C)**: diagnosis. Volcano plot represents the differences of **(D)**: osteoclast vs. monocyte, **(E)**: osteoclast vs. preosteoclast and **(F)**: preosteoclast vs. monocyte. Dashed horizontal line indicates adjusted p-value of 0.05.

First, we compared the samples using principal component analysis (PCA) dimensionality reduction to study which parameters have the most pronounced effect on the expression changes during differentiation. As shown in **Figure 2**, the clustering of the samples overlaps largely with the different stages of the differentiation. To quantify the effect of the differentiation on the clustering, we clustered the samples using the k-means clustering algorithm and the results with the sample labels using the adjusted Rand score index (32). Differentiation stage-based classification overlapped with the k-means clusters with a score of 0.89. On the other hand, neither the date of experiment, donor’s age, laboratory parameters (RF, anti-CCP, CRP, ESR), duration of disease, or DAS-28 score (**Supplementary Figure 1**) had any significant effect on the clustering of the samples. Our data show that the samples clearly separate from each other based on their cell type. The PCA analysis also suggests that there is a substantially bigger difference between the pre-osteoclasts (differentiation day 5) and monocytes (day 1) than between the pre-osteoclasts and osteoclasts (**Figure 2A**). This is also shown in **Figures 2D-F**, where the expression changes are plotted against the log p values. These findings are consistent with the osteoclast differentiation process, in which monocytes are gradually transformed first to preosteoclasts, and then to osteoclasts.

**Figure 3:**
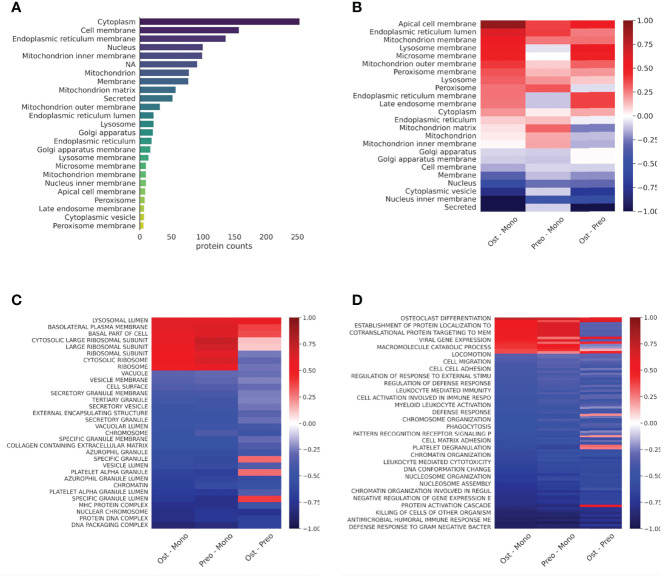
Protein distribution diverge in cell types in case of cellular location and protein expression in case of cell function. **(A)**: Bar graph representing the protein counts with different cellular locations. **(B)**: Heat map representing the protein expression differences of cell types in cellular location according to the Uniprot database. Average log2 differential expression data of all proteins localizing to given compartments were compared to overall average differential expression. **(C)**: Heat map representing the sample protein localizations, analyzed with GSE using GO: Cellular Compartments terms. **(D)**: Heat map representing the sample protein functions, analyzed with protein GSE using GO: Biological Process terms. For **(C)** and **(D)**, terms with the smallest adjusted p-values are shown on the heat-maps. Not all terms are indicated on the y-axis, the full table with p values are provided in **Supplementary Table 3**.

Next, we compared the localization information of the detected proteins of all samples using the Uniprot database. The proteins that were detected were localized primarily to the cytoplasm, cell membrane, endoplasmic reticulum membrane, nucleus, and mitochondria (**Figure 3A**). To gain an overall insight into the proteomic changes during the osteoclast development, we compared the changes in expression in three pairs of samples (osteoclasts vs. monocytes, pre-osteoclasts vs. monocytes, and osteoclasts vs. pre-osteoclasts). There are robust changes in the expression of proteins during the differentiation (**Figure 3**). Similar to the previously reported changes on the RAW264.7 cell line (33), there is increased expression of mitochondrial proteins, and decreased expression of nuclear proteins of osteoclasts compared to the monocytes (left lane). As expected from the transition of the non-polarized monocytes to polarized osteocytes, the expression of the apical proteins is enhanced (**Figure 3B**) (34, 35).

Next, we applied enrichment analysis to the differential expression data of all samples. Specifically, we compared the enrichment of the proteins based on their localizations (GO cellular compartment, GO-CC) and biological processes (GO-BP). As shown in **Figure 3** and **Supplementary Table 3**, expression of the different secretory vesicle proteins was decreased, while the basolateral membrane proteins were enriched in the osteoclast/preosteoclast group. Moreover, the proteins localized to the ribosomes were enriched during the later phase of the differentiation. In line with the transition from an immune cell to a bone-resorbing cell type, the expression of proteins in the MHC cellular compartment is also decreased. Furthermore, if we compare the changes in biological processes, the osteoclasts show enrichment of proteins involved in osteoclast differentiation and carbohydrate metabolism, while the processes involved in the immune functions of the monocytes, e.g. migration, cytokine production, and various proteins in immune responses are downregulated (**Figure 3** and **Supplementary Table 3**) (33, 36). The expression of basolateral proteins (besides apical proteins in **Figure 3B**) is enhanced as well (**Figure 3**). (30, 31).

**Figure 4:**
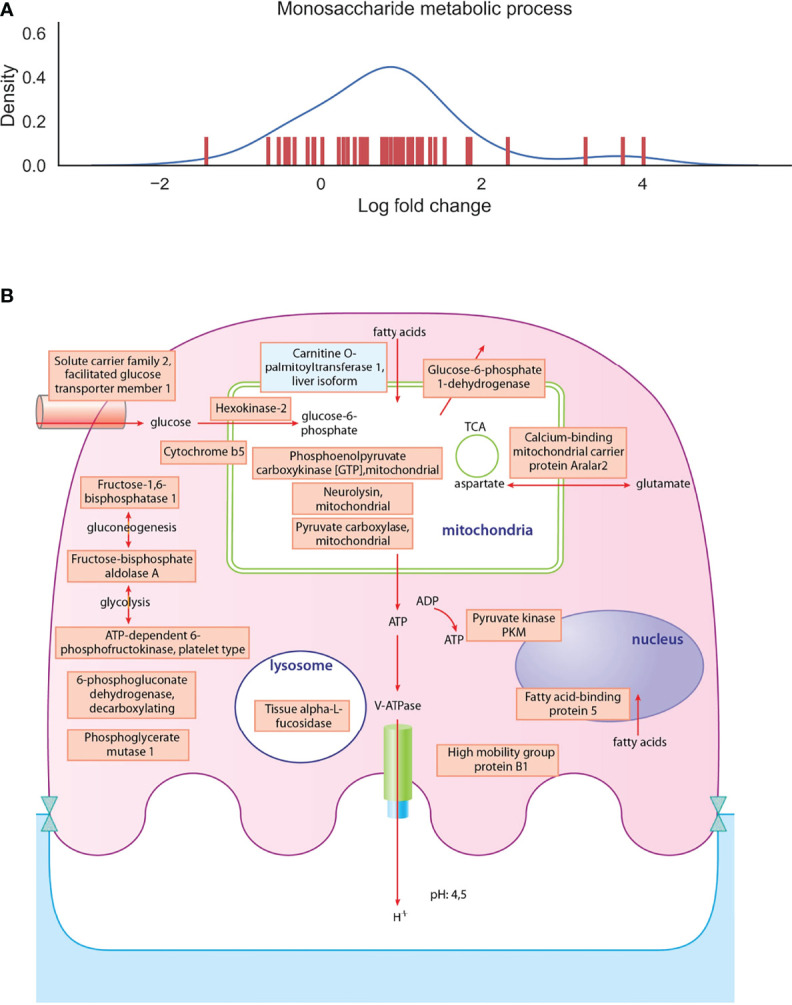
Depiction of proteins significantly expressed in osteoclasts. **(A)**: The density curve and individual protein differential expression log2 values of protein expressions in the monosaccharide metabolic process pathway identified in osteoclasts. **(B)**: drawing represents the most significantly expressed proteins involved in monosaccharide metabolic processes in osteoclast samples compared to monocyte samples. Blue filling shows downregulation of proteins in osteoclast samples compared to monocytes, red filling shows upregulation of proteins. The full table with p values are provided in **Supplementary Table 4**.

**Figure 5:**
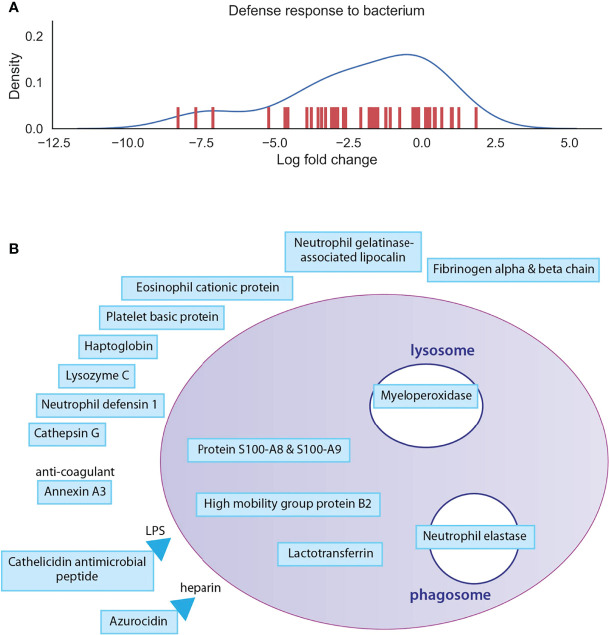
Depiction of proteins significantly expressed in monocytes. **(A)**: The density curve and individual protein differential expression log2 values of protein expressions in the defense response to bacterium pathway identified in osteoclasts. **(B)**: drawing represents the most significantly expressed proteins involved in defense response to bacterium in monocyte samples compared to osteoclast samples. Blue filling shows downregulation of proteins in osteoclast samples compared to monocytes. The full table with p values are provided in **Supplementary Table 4**.

To have a deeper look at the main differences between osteoclast and monocyte samples, we have selected two biological processes, one of which is enhanced and one of which is decreased), and depicted the involved proteins in **Figures 4**, **5** (**Supplementary Table 4**). Monosaccharide metabolic processes in osteoclast samples are increased in line with the elevated metabolic demands of these secretory cells (**Figures 4A**). On the other hand, proteins involved in the defense response to bacterium pathway are decreased in line with the transition during differentiation from monocytes into osteoclasts (**Figures 5A**). These include mainly secreted proteins, like cathepsin G and haptoglobin, but also some vesicular ones like myeloperoxidase.

Looking at the individual proteins, the biggest expression changes in the osteoclasts (over 10 fold differential expression changes) are connected to their transition from an immune cell to a cell type with a high level of metabolism and secretion and downregulated immune-function related proteins (**Supplementary Table 5**). The proteins with the highest differential expression in the osteoclasts compared to monocytes are mostly the ones that have been previously reported in the literature as some of the characteristic proteins in these cells. The increased metabolism is characterized by the overexpression of the *transferrin receptor protein 1* (required for mitochondrial respiration) (37). *Creatine kinase B-type* plays a central role in tissues with large energy, as it catalyzes the transfer of phosphate between phosphagens and ATP, and was found to be markedly induced in mature osteoclasts (19) and has a key role in osteoclast-mediated bone resorption (19, 38). The increased osteoclast-specific secretion is supported by the increased expression of vacuolar-type H^+^-ATPase (V-ATPase), *sodium/hydrogen exchanger 9B2* (a Na^+^/H^+^ antiporter) (39, 40), *cathepsin K and B* (proteolytic enzymes) (41, 42), and *lysosomal acid phosphatase* (43)*. Integrin alpha-V* crossing the cell membrane and anchored to the bone surface is essential, with which osteoclasts come into close contact with bone and promote the formation of the resorption lacunae (44). These results show, that the proteins with increased expression in osteoclasts detected in our experiments fit well with the previously reported characteristics of these cells.

Most of the proteins which are strongly downregulated in healthy osteoclasts play an important role in immune responses (e. g. *complement receptor type 1*), or in inflammatory processes (e. g. *protein S100-A8*), or have an antiviral (e. g. *neutrophil defensin 1*) or an antimicrobial (e. g. l*ysozyme C*) effect. *Platelet glycoprotein Ib beta chain* is important in blood coagulation, while *Annexin A3* is an anticoagulant. Some proteins (e. g. *intercellular adhesion molecule 3)* play a role in cell adhesion and *phospholipase B-like 1* in lipid metabolism. *Myeloid cell nuclear differentiation antigen* is a transcription regulator. Some proteins (e. g. *interferon-induced transmembrane protein 2)* have a role in innate immunity, some in adaptive immunity (e. g. *immunoglobulin kappa constant*), while *ficolin-1* in both (**Supplementary Table 5**).

### 3.2 Differences Between Healthy Control and Patient Samples Manifest During Osteoclast Development

**Figure 6:**
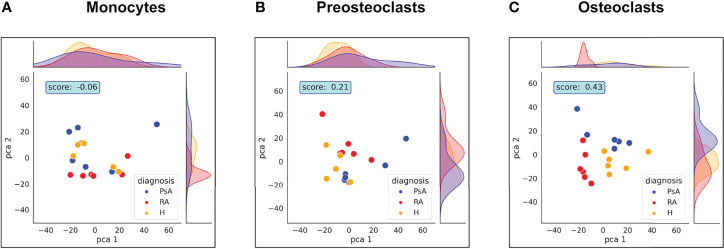
Healthy, RA or PsA samples vary mostly in osteoclast state. PCA analysis representing the differences between healthy, RA or PsA samples in **(A)** monocytes, **(B)** preosteoclasts and **(C)** osteoclasts.

In certain autoimmune diseases, like RA and PsA, activation of osteoclast cells is increased as a result of the inflammation, which promotes the production of TNF and RANK-L. Increased osteoclast activation fosters pathological bone destruction, such as the erosion of the bone and cartilage, leading to focal osteolysis of the inflamed joints. Therefore, we next investigated if there are differences in protein expression in osteoclasts originating from healthy subjects and those from patients with RA or PsA. As shown in **Figure 2C**, diagnoses did not have a significant impact on the clustering of the samples, when all cell types were included in the analysis. Therefore, we tested whether the differences are more pronounced if the samples are clustered within cell types. Surprisingly, as shown in **Figure 6**, we could detect separation of the cells with PCA analysis only when the cells were fully differentiated, but not when the monocytes were compared, suggesting that these diseases had overall more significant effects on the protein expressions of the osteoclasts than in monocytes.

The Rand index score generated by the cluster strength algorithm was increased during cell differentiation, reaching a 0.43 value in the case of osteoclast samples. Surprisingly, these findings suggest that when monocytes are isolated from the blood of healthy donors and RA or PsA patients and differentiated *in vitro* under the same circumstances, differences between healthy and unhealthy samples still manifest in more mature stages of cell development.

### 3.3 RA and PsA Osteoclasts Show Increased Expression of Proteins Involved in Immunological Processes

**Figure 7:**
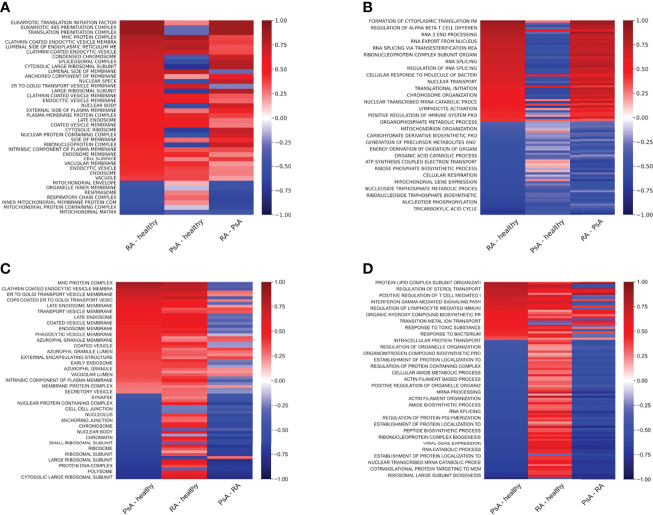
Protein expression differences of RA and PsA samples compared to healthy samples. Heat maps representing the sample protein localizations **(A, C)**, or functions **(B, D)** analyzed with protein GSE using GO: Cellular Compartments **(A, C)** and Biological Process terms **(B ,D)** for RA versus healthy **(A, B)**, or PsA versus healthy **(C, D)** samples. Terms with the smallest adjusted p-values are shown on the heat-map. Not all terms are indicated on the y-axis, the full table with p values are provided in **Supplementary Table 6**.

**Figure 8:**
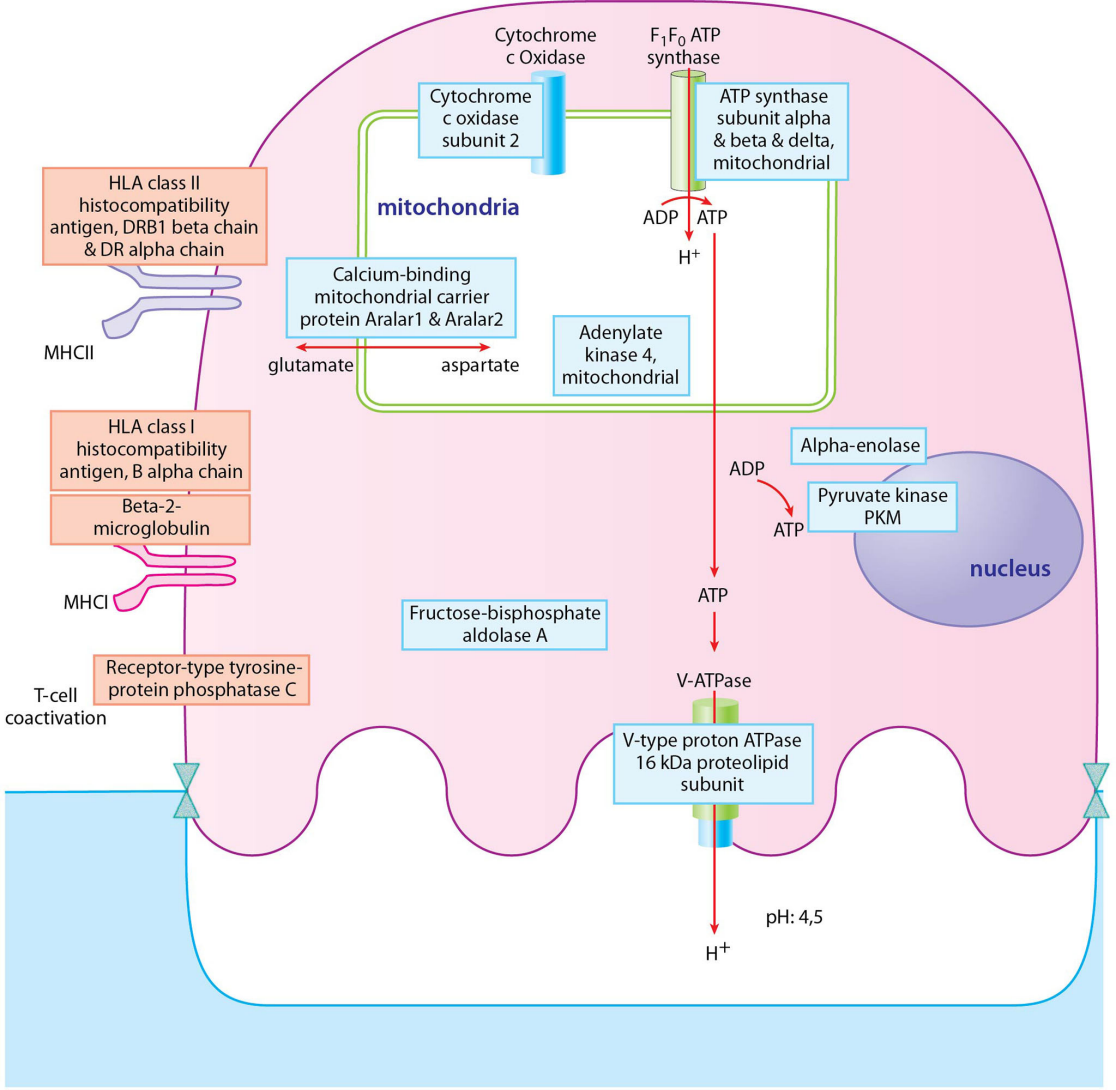
Depiction of proteins significantly expressed in osteoclasts of RA donors. Proteins with blue frame represent proteins of ATP biosynthetic processes pathway, proteins with red frame represent proteins of regulation of T cell mediated cytotoxicity pathway. Blue filling shows downregulation of proteins in RA osteoclast samples compared to healthy ones, red filling shows upregulation of proteins.

Since the largest differences between healthy and unhealthy samples were found in osteoclasts, we focused on these cells during the enrichment analysis of the RA and PsA samples. **Figure 7** and **Supplementary Table 6** show the enriched cellular compartments and biological processes, respectively, in which the RA samples significantly differ from the healthy ones. Interestingly, the RA and healthy osteoclasts show marked differences regarding to protein cellular compartments and biological processes. Regarding the compartments, most changes are related to different membrane compartments, most likely pointing to changes in vesicular trafficking (**Figure 7**). Interestingly, some of the proteins involved in the ATP biosynthetic processes were decreased, while proteins playing role in immunological processes were increased, like the antigen-presenting MHC-I and MHC-II proteins (**Figures 7**, **8**). These data suggest, that osteoclasts differentiated from the RA patients’ monocytes fail to fully adapt to the increased metabolic demand found in healthy osteoclasts and retain some of their immunological functions. One explanation of these proteome characteristics in RA osteoclast could be a different rate of differentiation, resulting in a higher proportion of undifferentiated cells at the end of the experiments. To explore this possibility, we have created a series of weighted protein level averages of osteoclasts and either monocytes or preosteoclasts to simulate a mixture of cells. When we have averaged these proteins from non-osteoclast samples with weights between 0 and 1 with those from osteoclasts (with a weight of 1), the clustering of the healthy and RA samples still clustered in different groups when checked with PCA analysis (**Figure 2**). This suggests that even if some of the differences might stem from the slower rate of differentiation in either of the samples, this does not fully explain the detected phenotypes.

**Figure 9:**
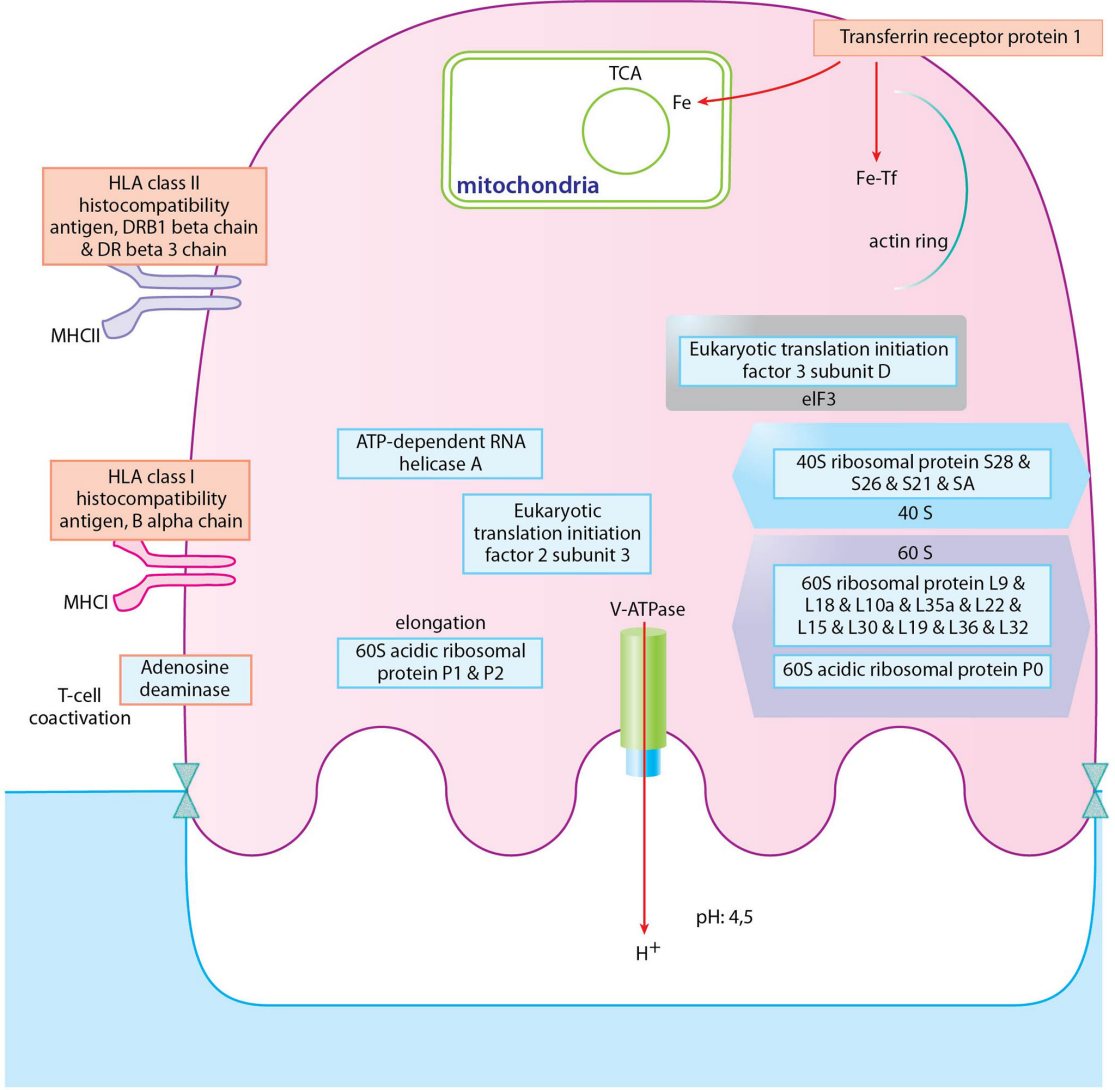
Depiction of proteins significantly expressed in osteoclasts of PsA donors. Proteins with blue frame represent proteins of cytoplasmic translation pathway, proteins with red frame represent proteins of positive regulation of adaptive immune response pathway. Blue filling shows downregulation of proteins in PsA osteoclast samples compared to healthy ones, red filling shows upregulation of proteins.

In the case of the PsA samples, proteins localized to Golgi, endosomes, several types of endocytic and phagocytic vesicles and granules, endoplasmatic reticulum, and MHC protein complexes (**Figure 7** and **Supplementary Table 6**). In addition, several processes involved in immunological functions, like antigen presentation, response to bacterium, positive regulation of adaptive immune response, and positive regulation of lymphocyte responses were increased in PsA samples (**Figures 7**, **9**). On the other hand, the expression of ribosomal, nuclear, and chromosome proteins, connected to organelle organization, metabolic processes, cytoskeleton, transcription, and translation was decreased (**Figures 7**, **9**).

## 4 Discussion

Mass spectrometry (MS)-based proteomics is widely used for the identification and quantification of proteins expressed in biological samples (15–21). Although in addition to the physiological bone remodeling, osteoclasts play a central role in the pathogenesis of both RA and PsA, the detailed *in vitro* proteomic analysis of osteoclastogenesis from monocytes was not investigated yet. Previous proteomic studies on the one hand investigated mice originating from BMM cultures, which can be differentiated into osteoclasts in 3 days (22). On the other hand, the majority of the murine studies focused on the RAW264.7 cell line, which is a monocyte/macrophage osteoclastogenesis model; can be committed to pre-osteoclasts in 2 days and to osteoclasts in 3 days (17). The proteomics of human blood-derived monocytes was also studied (without further differentiation to osteoclasts) (23–25).

Here for the first time, we report the proteomic changes during human blood-derived osteoclastogenesis of healthy donors’ as well as in RA and PsA. According to our present data, mitochondrial proteins were upregulated, whereas nuclear proteins, secreted proteins, and proteins involved in diverse immune functions were downregulated during osteoclastogenesis of healthy donor-derived monocytes (**Figure 3**). Eunkyung An et al. also found increased mitochondrial protein expression during the differentiation of RAW264.7 cells into osteoclasts (33). These findings are consistent with the high energy demand of bone resorption activity (33, 38). Interestingly, unlike monocytes, osteoclasts of healthy, RA, or PsA samples could be well discriminated based on their protein expression (**Figure 6**). Both in RA and PsA osteoclasts, the expression of proteins involved in immunological processes and proteins of the MHC complex was increased compared to healthy samples (**Figure 7**). The expression of proteins involved in ATP synthesis is decreased in RA osteoclasts, whereas processes involved in protein synthesis are downregulated in PsA samples. A decrease in protein synthesis might signal redirection of the cellular resources to other demanding processes, which in this case could be the enhanced immunological processes and intensive vesicular transports. The link between lipid oxidation and psoriasis was recently studied (45). Proteins involved in metabolic processes (e.g. *mitochondrial import receptor subunit TOM6 homolog*) were decreased in samples of both RA and PsA compared to healthy osteoclasts (**Figure 7**). The small number of healthy, RA, or PsA donors is a limitation in our present study. It has to be also noted, that the applied model in this experiment system does not fully model the native osteoclast differentiation when osteoclasts differentiate from local precursors, not from monocytes circulating in the blood.

RA and PsA may have similar symptoms and similarities in their pathomechanism. Serologic analysis shows the main differences between these two diseases; 70-80% of RA patients are seropositive for RF (rheumatoid factor) and/or anti-CCP (cyclic citrullinated peptide) autoantibody, while most of the PsA patients are RF and/or anti-CCP negative. Predisposing genetic factors also differ in these diseases; the presence of HLA-B27 and HLA-DR4 alleles predispose PsA, while the HLA-DRB1 allele (46) and single nucleotide polymorphism of the PTPN22 gene (47) predispose RA. There are also main differences in the development of pathology of these two diseases. In RA IL-1 and IL-6, RANK-L and ACPA (anti-citrullinated protein antibodies) are associated with the induction of osteoclastogenesis, and the number of T- and B-cells is higher in the inflamed synovial tissue in RA than in PsA (48–50). IL-17 and IL-23 cytokines in turn have a more dominant role in PsA disease development, and the number of T-helper 17 cells is higher in PsA than in RA (51). TNF-α inhibitors and JAK inhibitors are beneficial in both RA and PsA. Inhibition of IL-1 and IL-6 cytokines, B-cells or T-cells, is beneficial in RA; while inhibition of IL-12/23 and IL-17 have therapeutic efficacy in PsA (52). These differences in the cytokine environment might result in altered expression profiles in differentiated osteoclasts themself, or alternatively, might inhibit the differentiation process and lead to remaining immunological functions in these cells. Since the differences are more pronounced after differentiation, the latter option could seem more likely. However, when the protein levels of the osteoclasts are corrected with weighted averaging with those of the precursors, the clustering of the samples does not disappear, suggesting that the different rate of the differentiation itself does not explain all the differences detected. More likely, the monocytes derived from patients with RA or PsA differentiate to osteoclasts, but some of the antigen-presenting and other immune processes remain. Interestingly, it has been reported, that Hsp90α protein expression promotes osteoclast differentiation through regulation of the PPARγ pathway (53). In our samples, both Hsp90α and Hsp90β expressions were decreased, which is in agreement with the impaired transition of these cells from immune cells into osteoclasts. Nevertheless, in both RA and PsA, the bone erosions may be present, although in RA they mainly appear in peripheral joints and show symmetric involvement, while in PsA they may appear in both peripheral and axial joints and generally show asymmetric involvement. These bone erosions are in line with our results that suggest that osteoclasts in these diseases might keep some of their immune cell functions.

In summary, this is the first human study that examines the detailed proteomic changes during human osteoclast differentiation. Based on our results, protein expression differences between RA and PsA osteoclasts reveal immune and other functional differences in these diseases. Further studies with a higher number of patients including different disease stages (e.g. pre-RA or difficult-to-treat RA (54)) will provide future data regarding osteoclastogenesis in inflammatory arthropathies.

## Data Availability Statement

The original contributions presented in the study are publicly available. This data can be found here: https://doi.org/doi:10.25345/C5M61BT0F.

## Ethics Statement

The studies involving human participants were reviewed and approved by Ministry of Human Resources, Budapest, Hungary. The patients/participants provided their written informed consent to participate in this study.

## Author Contributions

LD, GN, GT, ET, and OK contributed to conception of the study. LD, ET, GT, NM, OK, and OO developed the methodology. ET, GT, OK, and OO performed formal analysis. ET, GT, OK, and OO performed data curation. GN, GT, and OK wrote the first draft of the manuscript. OK, ET, OO, NM, EB, LD, LH, GT, ESK and GN wrote sections of or edited the manuscript. GT and OK prepared the figures. GT, GN, and LH supervised the study. OK guided the project administration. LH, GT, and GN provided the funding ESK performed verification experiments. All authors contributed to manuscript revision, read, and approved the submitted version.

## Funding

This work was supported by grants from National Scientific Research Programs OTKA K-131479 and OTKA FK-138862, Rheuma Tolerance for Cure H2020 777357, and Competitive Central Hungary Operational Programme VEKOP-2.3.2-16-2016-00002. Project no. TKP-76-8/PALY-2021 has been implemented with the support provided by the Ministry of Innovation and Technology of Hungary from the National Research, Development, and Innovation Fund, financed under the TKP2021-EGA/TKP2021-NVA/TKP2021-NKTA funding scheme.

## Conflict of Interest

The authors declare that the research was conducted in the absence of any commercial or financial relationships that could be construed as a potential conflict of interest.

## Publisher’s Note

All claims expressed in this article are solely those of the authors and do not necessarily represent those of their affiliated organizations, or those of the publisher, the editors and the reviewers. Any product that may be evaluated in this article, or claim that may be made by its manufacturer, is not guaranteed or endorsed by the publisher.

## Supplementary Material

Supplementary Figure 1 Several parameters had no significant effect on the clustering of the samples. PCA analysis represent the clustering of samples based on **(A)**: rheumatoid factor (RF), **(B)**: cyclic citrullinated peptide (anti-CCP), **(C)**: C-reactive protein (CRP), and **(D)**: erythrocyte sedimentation rate (ESR) laboratory parameters, and **(E)**: DAS-28 score and **(F)**: duration of disease.

Supplementary Figure 2 In silico mixing of osteoclast proteins with monocyte or preosteoclast samples. PCA analysis of healthy and RA osteoclast proteins. Either healthy (middle row) or RA (bottom row) osteoclasts were mixed with monocyte or preosteoclast proteins with weights of 0.1 and 0.3 and compared to unmixed samples (upper left).

Supplementary Table 1 List of identified proteins with raw LFQ data. Table represents 1435 identified proteins across 18 samples.

Supplementary Table 2 List of identified proteins with imputed LFQ values. Table represents 1435 identified proteins across 18 samples.

Supplementary Table 3 GSE data. Table with data from **Figure 3C** and **D**.

Supplementary Table 4 GSE data. Proteins involved in monosaccharide metabolic process and defense response to bacterium pathways. Differential expression of osteoclasts and monocytes.

Supplementary Table 5 The biggest protein expression changes in osteoclasts. Table represents overexpressed or downregulated proteins (over 10 fold DE changes) in osteoclasts compared to monocytes.

Supplementary Table 6 GSE data. Table with data from **Figures 7A–D**.

## References

[1] Lemaire V Tobin FL Greller LD Cho CR Suva LJ. Modeling the Interactions Between Osteoblast and Osteoclast Activities in Bone Remodeling. J Theor Biol (2004) 229(3):293–309. doi: 10.1016/j.jtbi.2004.03.023 15234198

[2] Nakagawa N Kinosaki M Yamaguchi K Shima N Yasuda H Yano K. RANK is the Essential Signaling Receptor for Osteoclast Differentiation Factor in Osteoclastogenesis. Biochem Biophys Res Commun (1998) 253(2):395–400. doi: 10.1006/bbrc.1998.9788 9878548

[3] Asagiri M Takayanagi H. The Molecular Understanding of Osteoclast Differentiation. Bone (2007) 40(2):251–64. doi: 10.1016/j.bone.2006.09.023 17098490

[4] Miyamoto T. Regulators of Osteoclast Differentiation and Cell-Cell Fusion. Keio J Med (2011) 60(4):101–5. doi: 10.2302/kjm.60.101 22200633

[5] Hodge JM Kirkland MA Nicholson GC. Multiple Roles of M-CSF in Human Osteoclastogenesis. J Cell Biochem (2007) 102(3):759–68. doi: 10.1002/jcb.21331 17516513

[6] Bernhardt A Bacova J Gbureck U Gelinsky M. Influence of Cu2+ on Osteoclast Formation and Activity *In Vitro* . Int J Mol Sci (2021) 22(5). doi: 10.3390/ijms22052451 PMC7957576 33671069

[7] Sabokbar A Athanasou NS. Generating Human Osteoclasts From Peripheral Blood. Methods Mol Med (2003) 80:101–11. doi: 10.1385/1-59259-366-6:101 12728713

[8] Miyamoto T Arai F Ohneda O Takagi K Anderson DM Suda T. An Adherent Condition is Required for Formation of Multinuclear Osteoclasts in the Presence of Macrophage Colony-Stimulating Factor and Receptor Activator of Nuclear Factor Kappa B Ligand. Blood (2000) 96(13):4335–43. doi: 10.1182/blood.V96.13.4335 11110710

[9] Pánczél Á Nagy SP Farkas J Jakus Z Győri DS Mócsai A. Fluorescence-Based Real-Time Analysis of Osteoclast Development. Front Cell Dev Biol (2021) 9:657935. doi: 10.3389/fcell.2021.657935 34327196 PMC8314002

[10] Csete D Simon E Alatshan A Aradi P Dobó-Nagy C Jakus Z. Hematopoietic or Osteoclast-Specific Deletion of Syk Leads to Increased Bone Mass in Experimental Mice. Front Immunol (2019) 10:937. doi: 10.3389/fimmu.2019.00937 31134061 PMC6524727

[11] Rössler U Hennig AF Stelzer N Bose S Kopp J Søe K. Efficient Generation of Osteoclasts From Human Induced Pluripotent Stem Cells and Functional Investigations of Lethal CLCN7-Related Osteopetrosis. J Bone Miner Res (2021) 36(8):1621–35. doi: 10.1002/jbmr.4322 33905594

[12] Yokota K Sato K Miyazaki T Aizaki Y Tanaka S Sekikawa M. Characterization and Function of Tumor Necrosis Factor and Interleukin-6-Induced Osteoclasts in Rheumatoid Arthritis. Arthritis Rheumatol (2021) 73(7):1145–54. doi: 10.1002/art.41666 PMC8361923 33512089

[13] Walsh MC Choi Y. Biology of the TRANCE Axis. Cytokine Growth Factor Rev (2003) 14(3-4):251–63. doi: 10.1016/s1359-6101(03)00027-3 12787563

[14] Paine A Ritchlin C. Bone Remodeling in Psoriasis and Psoriatic Arthritis: An Update. Curr Opin Rheumatol (2016) 28(1):66–75. doi: 10.1097/BOR.0000000000000232 26555451

[15] Heckel T Czupalla C Expirto Santo AI Anitei M Arantzazu Sanchez-Fernandez M Mosch K. Src-Dependent Repression of ARF6 is Required to Maintain Podosome-Rich Sealing Zones in Bone-Digesting Osteoclasts. Proc Natl Acad Sci U S A (2009) 106(5):1451–6. doi: 10.1073/pnas.0804464106 PMC2635769 19164586

[16] Ryu J Kim H Lee SK Chang E-J Kim HJ Kim H-H. Proteomic Identification of the TRAF6 Regulation of Vacuolar ATPase for Osteoclast Function. Proteomics (2005) 5(16):4152–60. doi: 10.1002/pmic.200402035 16196101

[17] Chang E-J Kwak HB Kim H Park J-C Lee ZH Kim H-H. Elucidation of CPX-1 Involvement in RANKL-Induced Osteoclastogenesis by a Proteomics Approach. FEBS Lett (2004) 564(1-2):166–70. doi: 10.1016/S0014-5793(04)00338-2 15094061

[18] Ha BG Hong JM Park J-Y Ha M-H Kim T-H Cho J-Y. Proteomic Profile of Osteoclast Membrane Proteins: Identification of Na+/H+ Exchanger Domain Containing 2 and its Role in Osteoclast Fusion. Proteomics (2008) 8(13):2625–39. doi: 10.1002/pmic.200701192 18600791

[19] Chen J Sun Y Mao X Liu Q Wu H Chen Y. RANKL Up-Regulates Brain-Type Creatine Kinase *via* Poly(ADP-Ribose) Polymerase-1 During Osteoclastogenesis. J Biol Chem (2010) 285(47):36315–21. doi: 10.1074/jbc.M110.157743 PMC2978559 20837480

[20] Manes NP Angermann BR Koppenol-Raab M An E Sjoelund VH Sun J. Targeted Proteomics-Driven Computational Modeling of Macrophage S1P Chemosensing. Mol Cell Proteomics (2015) 14(10):2661–81. doi: 10.1074/mcp.M115.048918 PMC4597143 26199343

[21] Freire MS Cantuária APC Lima SMF Almeida JA Murad AM Franco OL. NanoUPLC-MS(E) Proteomic Analysis of Osteoclastogenesis Downregulation by IL-4. J Proteomics (2016) 131:8–16. doi: 10.1016/j.jprot.2015.10.004 26459402

[22] Ryu J Kim H Chang E-J Kim HJ Lee Y Kim H-H. Proteomic Analysis of Osteoclast Lipid Rafts: The Role of the Integrity of Lipid Rafts on V-ATPase Activity in Osteoclasts. J Bone Miner Metab (2010) 28(4):410–7. doi: 10.1007/s00774-009-0150-y 20127130

[23] Zeng Y Zhang L Zhu W Xu C He H Zhou Y. Quantitative Proteomics and Integrative Network Analysis Identified Novel Genes and Pathways Related to Osteoporosis. J Proteomics (2016) 142:45–52. doi: 10.1016/j.jprot.2016.04.044 27153759 PMC5362378

[24] Daswani B Gupta MK Gavali S Desai M Sathe GJ Patil A. Monocyte Proteomics Reveals Involvement of Phosphorylated HSP27 in the Pathogenesis of Osteoporosis. Dis Markers (2015) 2015:196589. doi: 10.1155/2015/196589 26063949 PMC4439496

[25] Zhang L Liu Y-Z Zeng Y Zhu W Zhao Y-C Zhang J-G. Network-Based Proteomic Analysis for Postmenopausal Osteoporosis in Caucasian Females. Proteomics (2016) 16(1):12–28. doi: 10.1002/pmic.201500005 26435169

[26] Aletaha D Neogi T Silman AJ Funovits J Felson DT Bingham CO 3rd. 2010 Rheumatoid Arthritis Classification Criteria: An American College of Rheumatology/European League Against Rheumatism Collaborative Initiative. Ann Rheum Dis (2010) 69(9):1580–8. doi: 10.1136/ard.2010.138461 20699241

[27] Taylor W Gladman D Helliwell P Marchesoni A Mease P Mielants H. Classification Criteria for Psoriatic Arthritis: Development of New Criteria From a Large International Study. Arthritis Rheum (2006) 54(8):2665–73. doi: 10.1002/art.21972 16871531

[28] Rickham PP. Human experimentation. Code of ethics of the world medical association. Declaration of helsinki. Br Med J (1964) 2(5402):177. doi: 10.1136/bmj.2.5402.177 14150898 PMC1816102

[29] MacLellan LM Montgomery J Sugiyama F Kitson SM Thummler K Silverman GJ. Co-Opting Endogenous Immunoglobulin for the Regulation of Inflammation and Osteoclastogenesis in Humans and Mice. Arthritis Rheum (2011) 63(12):3897–907. doi: 10.1002/art.30629 PMC3598489 22127707

[30] Steinley D. Properties of the Hubert-Arabie Adjusted Rand Index. Psychol Methods (2004) 9(3):386–96. doi: 10.1037/1082-989X.9.3.386 15355155

[31] Marton N Kovács OT Baricza E Kittel Á Győri D Mócsai A. Extracellular Vesicles Regulate the Human Osteoclastogenesis: Divergent Roles in Discrete Inflammatory Arthropathies. Cell Mol Life Sci (2017) 74(19):3599–611. doi: 10.1007/s00018-017-2535-8 PMC11107760 28493076

[32] Saryan P Gupta S Gowda V. Species Complex Delimitations in the Genus Hedychium: A Machine Learning Approach for Cluster Discovery. Appl Plant Sci (2020) 8(7):e11377. doi: 10.1002/aps3.11377 32765976 PMC7394710

[33] An E Narayanan M Manes NP Nita-Lazar A. Characterization of Functional Reprogramming During Osteoclast Development Using Quantitative Proteomics and mRNA Profiling. Mol Cell Proteomics (2014) 13(10):2687–704. doi: 10.1074/mcp.M113.034371 PMC4188996 25044017

[34] Baron R. Polarity and Membrane Transport in Osteoclasts. Connect Tissue Res (1989) 20(1-4):109–20. doi: 10.3109/03008208909023879 2692952

[35] Inui T Ishibashi O Origane Y Fujimori K Kokubo T Nakajima M. Matrix Metalloproteinases and Lysosomal Cysteine Proteases in Osteoclasts Contribute to Bone Resorption Through Distinct Modes of Action. Biochem Biophys Res Commun (1999) 258(1):173–8. doi: 10.1006/bbrc.1999.0473 10222255

[36] Bordbar A Mo ML Nakayasu ES Schrimpe-Rutledge AC Kim Y-M Metz TO. Model-Driven Multi-Omic Data Analysis Elucidates Metabolic Immunomodulators of Macrophage Activation. Mol Syst Biol (2012) 8:558. doi: 10.1038/msb.2012.21 22735334 PMC3397418

[37] Xie W Lorenz S Dolder S Hofstetter W. Extracellular Iron is a Modulator of the Differentiation of Osteoclast Lineage Cells. Calcif Tissue Int (2016) 98(3):275–83. doi: 10.1007/s00223-015-0087-1 26615413

[38] Chang E-J Ha J Oerlemans F Lee YJ Lee SW Ryu J. Brain-Type Creatine Kinase has a Crucial Role in Osteoclast-Mediated Bone Resorption. Nat Med (2008) 14(9):966–72. doi: 10.1038/nm.1860 18724377

[39] Rousselle A-V Heymann D. Osteoclastic Acidification Pathways During Bone Resorption. Bone (2002) 30(4):533–40. doi: 10.1016/s8756-3282(02)00672-5 11934642

[40] Hall TJ Chambers TJ. Na+/H+ Antiporter is the Primary Proton Transport System Used by Osteoclasts During Bone Resorption. J Cell Physiol (1990) 142(2):420–4. doi: 10.1002/jcp.1041420227 2154508

[41] Dai R Wu Z Chu HY Lu J Lyu A Liu J. Cathepsin K: The Action in and Beyond Bone. Front Cell Dev Biol (2020) 8:433. doi: 10.3389/fcell.2020.00433 32582709 PMC7287012

[42] Delaissé JM Ledent P Vaes G. Collagenolytic Cysteine Proteinases of Bone Tissue. Cathepsin B, (Pro)Cathepsin L and a Cathepsin L-Like 70 kDa Proteinase. Biochem J (1991) 279(Pt 1):167–74. doi: 10.1042/bj2790167 PMC1151563 1930136

[43] Ohsawa Y Nitatori T Higuchi S Kominami E Uchiyama Y. Lysosomal Cysteine and Aspartic Proteinases, Acid Phosphatase, and an Endogenous Cysteine Proteinase Inhibitor, Cystatin-Beta, in Rat Osteoclasts. J Histochem Cytochem (1993) 41(7):1075–83. doi: 10.1177/41.7.8515049 8515049

[44] Xiang B Liu Y Zhao W Zhao H Yu H. Extracellular Calcium Regulates the Adhesion and Migration of Osteoclasts *via* Integrin αv β 3 /Rho A/Cytoskeleton Signaling. Cell Biol Int (2019) 43(10):1125–36. doi: 10.1002/cbin.11033 30022569

[45] Sorokin AV Remaley AT Mehta NN. Oxidized Lipids and Lipoprotein Dysfunction in Psoriasis. J Psoriasis Psoriatic Arthritis (2020) 5(4):139–46. doi: 10.1177/2475530320950268 PMC7646705 33163854

[46] Saalfeld W Mixon AM Zelie J Lydon EJ. Differentiating Psoriatic Arthritis From Osteoarthritis and Rheumatoid Arthritis: A Narrative Review and Guide for Advanced Practice Providers. Rheumatol Ther (2021) 8(4):1493–517. doi: 10.1007/s40744-021-00365-1 PMC8572231 34519965

[47] Abbasifard M Imani D Bagheri-Hosseinabadi Z. PTPN22 Gene Polymorphism and Susceptibility to Rheumatoid Arthritis (RA): Updated Systematic Review and Meta-Analysis. J Gene Med (2020) 22(9):e3204. doi: 10.1002/jgm.3204 32333475

[48] Takayanagi H. Osteoimmunology and the Effects of the Immune System on Bone. Nat Rev Rheumatol (2009) 5(12):667–76. doi: 10.1038/nrrheum.2009.217 19884898

[49] Harre U Georgess D Bang H Bozec A Axmann R Ossipova E. Induction of Osteoclastogenesis and Bone Loss by Human Autoantibodies Against Citrullinated Vimentin. J Clin Invest (2012) 122(5):1791–802. doi: 10.1172/JCI60975 PMC3336988 22505457

[50] Szarka E Babos F Magyar A Huber K Szittner Z Papp K. Recognition of New Citrulline-Containing Peptide Epitopes by Autoantibodies Produced *In Vivo* and *In Vitro* by B Cells of Rheumatoid Arthritis Patients. Immunology (2014) 141(2):181–91. doi: 10.1111/imm.12175 PMC3904239 24116744

[51] Baricza E Marton N Királyhidi P Kovács OT Kovácsné Székely I Lajkó E. Distinct *In Vitro* T-Helper 17 Differentiation Capacity of Peripheral Naive T Cells in Rheumatoid and Psoriatic Arthritis. Front Immunol (2018) 9:606. doi: 10.3389/fimmu.2018.00606 29670615 PMC5893718

[52] Mc Ardle A Flatley B Pennington SR FitzGerald O. Early Biomarkers of Joint Damage in Rheumatoid and Psoriatic Arthritis. Arthritis Res Ther (2015) 17:141. doi: 10.1186/s13075-015-0652-z 26028339 PMC4450469

[53] Ma J Yang C Zhong H Wang C Zhang K Li X. Role of HSP90α in Osteoclast Formation and Osteoporosis Development. Exp Ther Med (2022) 23(4):273. doi: 10.3892/etm.2022.11199 35251339 PMC8892609

[54] Nagy G Roodenrijs NM Welsing PM Kedves M Hamar A van der Goes MC. EULAR Definition of Difficult-to-Treat Rheumatoid Arthritis. Ann Rheum Dis (2021) 80(1):31–5. doi: 10.1136/annrheumdis-2020-217344 PMC7788062 33004335

