# Supplementary figures and images for: Proteomic Changes of Osteoclast Differentiation in Rheumatoid and Psoriatic Arthritis Reveal Functional Differences
Source: Front Immunol. 2022 Jul 4;13:892970. doi: 10.3389/fimmu.2022.892970 (PMC9289121; doi:10.3389/fimmu.2022.892970)

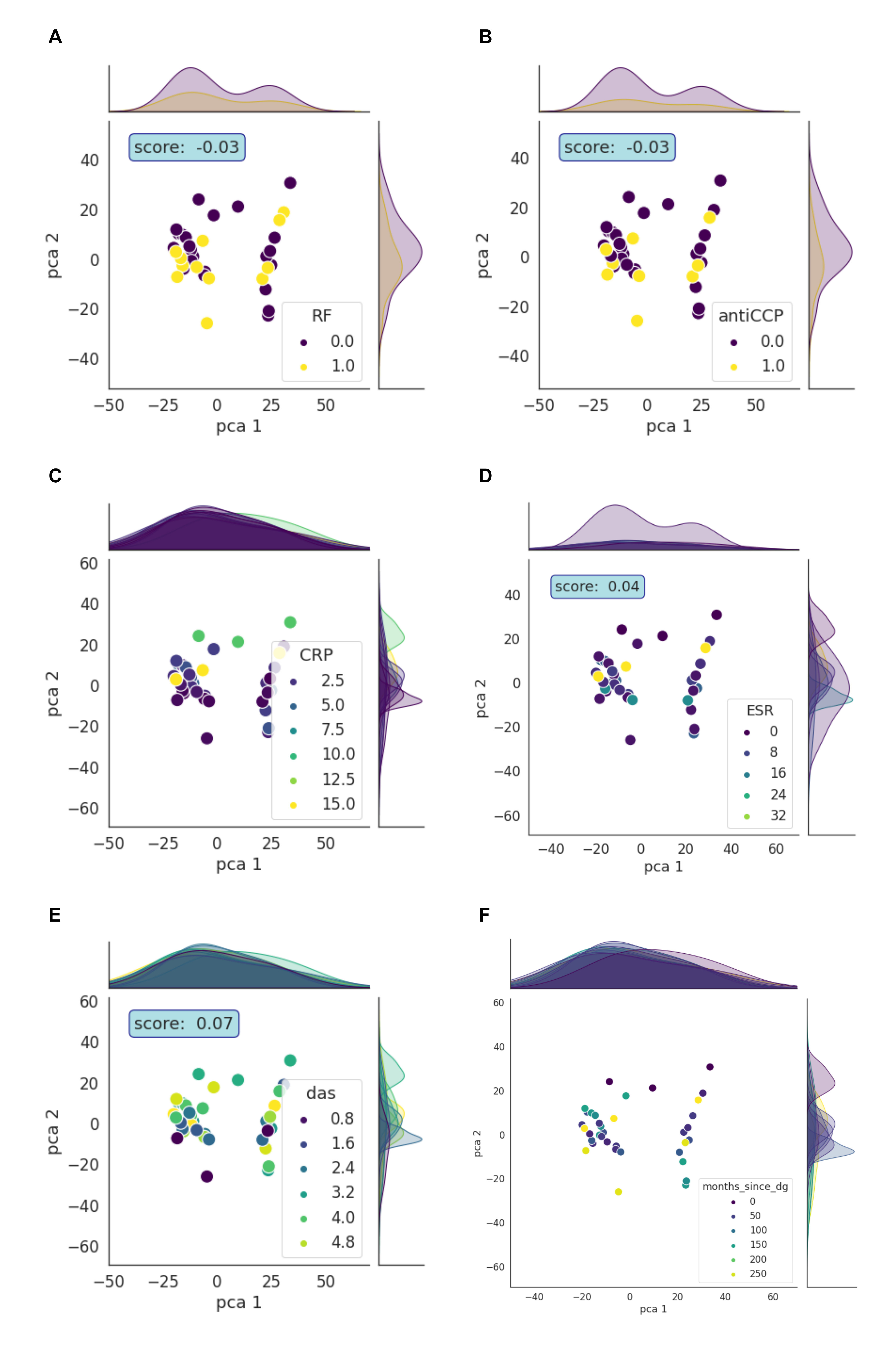

Supplement: Supplementary Figure 1 — Several parameters had no significant effect on the clustering of the samples. PCA analysis represent the clustering of samples based on (A): rheumatoid factor (RF), (B): cyclic citrullinated peptide (anti-CCP), (C): C-reactive protein (CRP), and (D): erythrocyte sedimentation rate (ESR) laboratory parameters, and (E): DAS-28 score and (F): duration of disease. [file Image_1.jpeg]

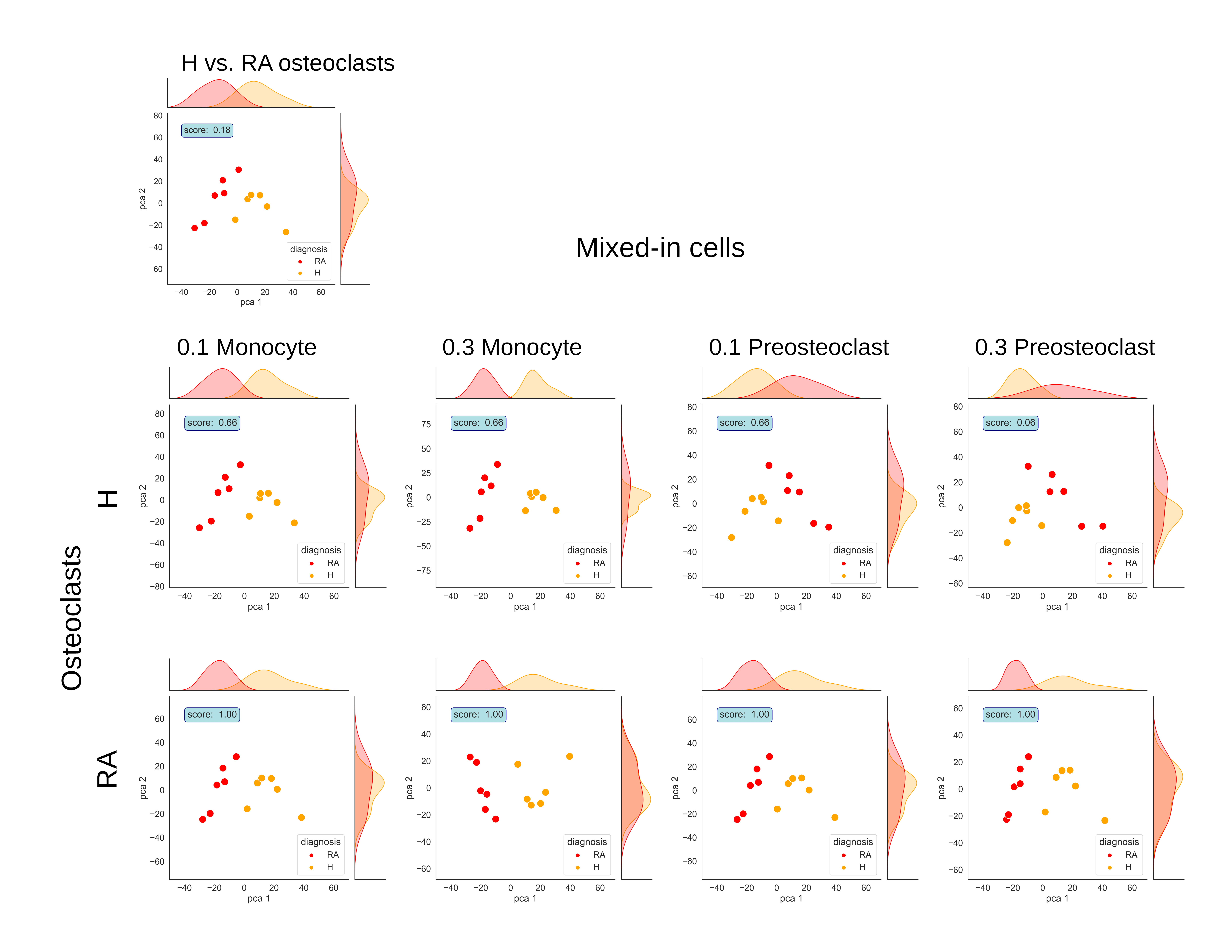

Supplement: Supplementary Figure 2 — In silico mixing of osteoclast proteins with monocyte or preosteoclast samples. PCA analysis of healthy and RA osteoclast proteins. Either healthy (middle row) or RA (bottom row) osteoclasts were mixed with monocyte or preosteoclast proteins with weights of 0.1 and 0.3 and compared to unmixed samples (upper left). [file Image_2.jpeg]
